# Porcine Models of Heart Regeneration

**DOI:** 10.3390/jcdd9040093

**Published:** 2022-03-23

**Authors:** Nivedhitha Velayutham, Katherine E. Yutzey

**Affiliations:** 1 The Heart Institute, Cincinnati Children’s Hospital Medical Center, Cincinnati, OH 45229, USA; nivedhitha.velayutham@cchmc.org; 2 Molecular and Developmental Biology Graduate Program, University of Cincinnati College of Medicine, Cincinnati, OH 45267, USA; 3 Department of Pediatrics, University of Cincinnati College of Medicine, Cincinnati, OH 45267, USA

**Keywords:** cardiac regeneration, pig heart, large mammal, cardiac preclinical studies

## Abstract

Swine are popular large mammals for cardiac preclinical testing due to their similarities with humans in terms of organ size and physiology. Recent studies indicate an early neonatal regenerative capacity for swine hearts similar to small mammal laboratory models such as rodents, inspiring exciting possibilities for studying cardiac regeneration with the goal of improved clinical translation to humans. However, while swine hearts are anatomically similar to humans, fundamental differences exist in growth mechanisms, nucleation, and the maturation of pig cardiomyocytes, which could present difficulties for the translation of preclinical findings in swine to human therapeutics. In this review, we discuss the maturational dynamics of pig cardiomyocytes and their capacity for proliferative cardiac regeneration during early neonatal development to provide a perspective on swine as a preclinical model for developing cardiac gene- and cell-based regenerative therapeutics.

## 1. Introduction

Heart failure is a highly prevalent disease in humans with no effective long-term treatment strategy beyond organ transplantation [1]. Loss of cardiomyocytes during progression of disease and the inability of adult human cardiomyocytes to sufficiently proliferate to restore lost muscle underlie the progression and lethality of heart disease. A regenerative cure for heart disease via the renewal of cardiomyocytes is thus an attractive therapeutic strategy to heal the failing heart.

Swine are widely utilized as a cardiac preclinical model, especially for testing surgical and mechanical interventions for heart disease [2]. At a gross anatomic level, human and pig hearts are very similar, and the comparable size makes pigs an attractive large mammal model for cardiovascular disease. However, at a cellular level, cardiomyocyte proliferation and growth mechanisms in swine differ considerably from other mammals [3], and this may hinder the clinical translation of findings in pigs to human cardiovascular disease. Thus, consideration of cardiac regenerative mechanisms in swine, along with the assessment of the advantages and disadvantages of the porcine model, is necessary for developing novel cardiac regenerative therapies.

Cardiac regeneration is the formation of new muscle to replace damaged muscle, via the proliferation of cardiomyocytes with minimal fibrotic scarring. While adult human heart regeneration remains elusive, the mechanisms underlying the regenerative potential of the heart can be elucidated by studying laboratory and preclinical model systems. Nonmammalian ectothermic vertebrates, such as zebrafish, newts, and axolotls, can regenerate their hearts after injury via cardiomyocyte proliferation throughout life, but adult endothermic mammals do not have this capacity [4]. However, a transient neonatal regenerative capacity, where new cardiomyocytes are produced via the proliferation of existing cardiomyocytes to regeneratively repair the heart, has been demonstrated in both neonatal mice and pigs in the first few days after birth [5,6,7].

In humans, functional recovery following myocardial infarction soon after birth has been described anecdotally, hinting at a potential for early neonatal heart regenerative capacity [8,9]. Limited studies of early neonatal human cardiac development showed cardiomyocyte mitotic activity, including the expression of cytokinesis markers, in the first months after birth [10,11]. However, the exact time of human cardiomyocyte terminal mitotic arrest after birth remains unclear. Ultimately, adult human cardiomyocytes have less than ~1% turnover per year [12], which is insufficient for regenerative healing after injury, such as myocardial infarction.

Studying porcine models of heart regeneration could offer insight into human postnatal cardiac regenerative potential and facilitate development of effective therapeutics for human cardiac regenerative repair. As a large mammal model, juvenile pigs (2–6 postnatal months) have been frequently utilized to test cardiac regenerative therapies for clinical translation. However, differences in cardiomyocyte growth characteristics in swine, such as extensive multinucleation and ongoing cardiomyocyte mitotic activity extending well into adolescence could be distinctive to pigs [3], which may influence the outcomes of such studies. In this review, we discuss pig cardiomyocyte maturation and proliferative activity during postnatal development in the context of using swine as a preclinical model for studying mammalian cardiac regeneration and repair.

## 2. Cardiac Maturation and Regenerative Potential in Neonatal Swine

### 2.1. Cardiac Maturational Mechanisms in Postnatal Pig Hearts

Pig hearts undergo rapid growth to nearly triple in size in the first month after birth [3]. Pig cardiomyocytes are predominantly mononucleated and diploid at birth. However, by postnatal day (P)7 to P14, there is an onset of rapid binucleation, followed by progressive multinucleation by one postnatal month and beyond. There is also robust mitotic activity at birth that is maintained in multinucleated cardiomyocytes for at least two postnatal months in swine. Further, gap junctional maturation and a switch to adult contractile protein isoforms do not occur in pig cardiomyocytes until more than 30 days after birth. These maturational mechanisms are significantly different from rodents, in which cardiomyocyte binucleation, terminal maturation, and the switch to hypertrophic growth all occur in the first few days after birth.

**Figure 1:**
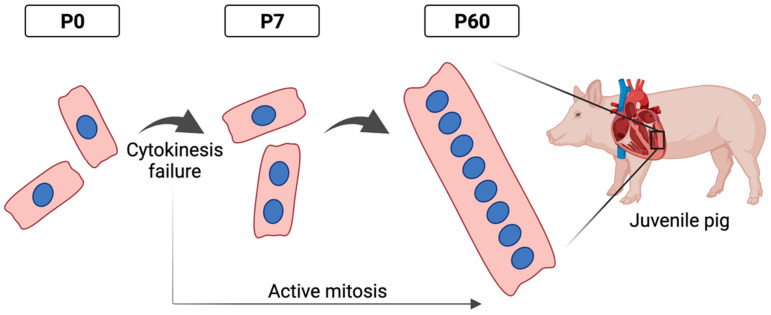
Schematic of transition to predominantly multinucleated cardiomyocyte state during postnatal development in swine. Juvenile pigs exhibit 4 to 16 nuclei per cardiomyocyte.

Pig cardiomyocytes grow longitudinally relative to nucleation before transitioning to a diametric mode of hypertrophic growth at 4–6 postnatal months, when cell cycle withdrawal finally occurs. However, cytokinetic arrest occurs in the first week after birth in porcine cardiomyocytes, similar to rodents, with most of the persisting mitotic activity resulting in extensive multinucleation. Pig cardiomyocytes exhibit four or more nuclei per cardiomyocyte beyond two postnatal weeks, and about 60–90% of cardiomyocytes are multinucleated in juvenile pigs that are commonly utilized for preclinical cardiac studies (Figure 1). These data show that swine are unique in that they exhibit prevalent acytokinetic mitosis of nuclei to generate highly multinucleated cardiomyocytes by a few weeks after birth. However, pigs are also similar to other mammals in that the proliferation of mononucleated cells to generate new cardiomyocytes ceases in the first few days after birth.

### 2.2. Transient Cardiac Regenerative Potential in Neonatal Pig Hearts

Mammalian cardiac regeneration was first reported in mice where regenerative repair without scarring was observed when cardiac injury occurred before P7, but injury in older mice resulted in scar formation and compromised heart function [5]. Similarly, pigs have a transient neonatal cardiac regenerative capacity upon injury within 2–3 days after birth [6,7]. Myocardial infarction (MI) at P2–3 induces cardiomyocyte proliferation leading to repair in the pig heart, with healthy new muscle and minimal fibrotic scarring by 30 days post-injury. However, cardiac injury beyond P3, at P7, P14 [6,7], or P30 [13], leads to extensive scarring by 30 days post-injury, with no functional recovery as well as reduced ventricular wall thickness. A follow-up study assessed the effect of apical resection at P1 on prolonging regenerative healing in neonatal pig hearts upon re-injury at P28 by MI [14]. This study found prolonged cell cycle activity and increased repair with MI-injury following apical resection, compared to an MI-only cohort. This suggests that hyperplastic growth induced by apical resection at P1 is still maintained at P28 and contributes to proliferative healing upon re-injury in late neonatal swine. Unlike mice and zebrafish, the extent of regenerative remuscularization following apical resection has not yet been systematically characterized in neonatal pig hearts.

Altogether, these reports indicate that there exists a shared early neonatal window of innate cardiac regenerative capacity for the first few days after birth in pigs and mice. During this time, injury to the heart results in regenerative healing and functional recovery without scar formation. However, in both model systems, this ability is lost beyond the first few days after birth. Comparing postnatal maturational and regenerative mechanisms between the model systems could facilitate an understanding of key processes that regulate mammalian cardiac regenerative potential.

### 2.3. Comparison of Cardiomyocyte Maturation and Regenerative Potential between Small and Large Mammal Model Systems

Some of the hallmark characteristics of terminal cardiomyocyte maturation as determined in rodents are: cardiomyocyte mitotic arrest, switch to hypertrophic mode of growth via increased cardiomyocyte cross-sectional size, sarcomeric maturation with switch to adult contractile protein isoforms, and a decline of mononucleated-diploid cardiomyocytes [15,16]. These events occur concurrent with the loss of regenerative potential in rodents, and thus have been considered as markers of regenerative potential in other species. However, the timing of some of these maturational events may not be directly comparable to innate cardiomyocyte proliferative capacity among the different mammalian species.

A significant presence of mononucleated-diploid cardiomyocytes has been highlighted as imperative for cardiomyocyte proliferation and hyperplastic growth during early postnatal development in rodents. In addition, cardiac regenerative capacity in rodents is dependent on the presence of a proliferative mononucleated-diploid cardiomyocyte population [17]. Further, evolutionary endothermy acquisition is linked to the loss of mononucleated-diploid cardiomyocytes in adult mammals, with a lack of heart regenerative capacity implicated as a consequence [18]. At P2–3, when porcine hearts are capable of regenerative repair, pig cardiomyocytes are predominantly mononucleated and diploid with active cell cycling. However, pig hearts retain nearly 50% mononucleated-diploid cardiomyocytes and active cell cycling up to P15, but form a fibrotic scar with no new muscle generation when infarcted at this age. Thus, there exists a discordance between cardiomyocyte terminal maturation and the loss of cardiac regenerative capacity in swine. Interestingly, anterior ventricular wall systolic thinning was comparatively less severe in pig cohorts infarcted at P7 [7], when nearly 75% mononucleated-diploid and mitotically active cardiomyocytes are still present in the porcine heart. This suggests some capacity for functional repair up to this stage and a potential threshold level of mononucleated-diploid actively cycling cardiomyocytes to be present for regenerative versus fibrotic healing after injury.

In neonatal pigs, while mitotic activity remains high at P7, cytokinesis-specific genes are downregulated at P7 compared to P0 by RNA analysis [3]. Interestingly, both *Ect2* (Epithelial Cell Transforming 2) and *Lmnb2* (Lamin B2) were significantly downregulated by P7 in pig hearts. Both genes are critical for successful proliferation, the depletion of which results in the onset of polyploidy in rodent cardiomyocytes [19,20]. Thus, cytokinetic factors that control the final stages of cell division could be promising targets for inducing cardiomyocyte proliferative activity across mammalian species. Other mechanistic pathways that were significantly differentially regulated by P7-P15 in swine compared to P0 were reactive oxygen species response pathways, extracellular matrix organization, sympathetic innervation, and innate immune system maturation [3], all of which have been linked to postnatal rodent regenerative potential. Further, during neonatal porcine heart regeneration, the upregulation of pathways for cell survival, proliferation, and angiogenesis was reported by transcriptomic analyses [6,7], similar to regenerative repair mechanisms in rodents.

**Figure 2:**
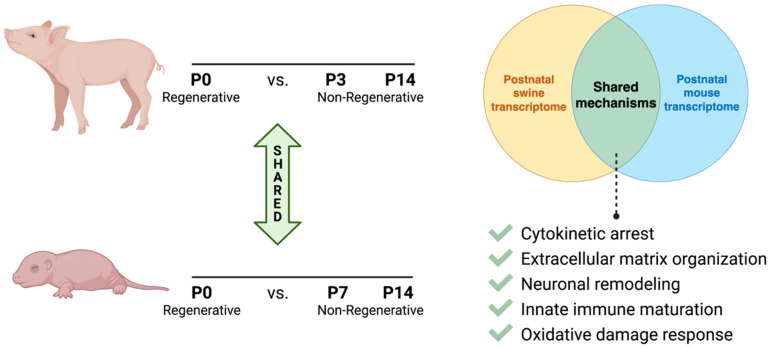
Schematic comparison of shared maturational mechanisms during neonatal loss of cardiac regenerative capacity in swine and rodents.

Studying such shared processes, including via the direct comparison of existing sequencing datasets during neonatal development, could facilitate the identification of new target genes and pathways critical for cardiomyocyte proliferation and heart regeneration (Figure 2). Understanding postnatal cardiomyocyte maturational dynamics in various mammalian systems will thus help to elucidate conserved mechanisms of mammalian heart regeneration.

## 3. Preclinical Strategies for Heart Regeneration in Juvenile Swine

**Figure 3:**
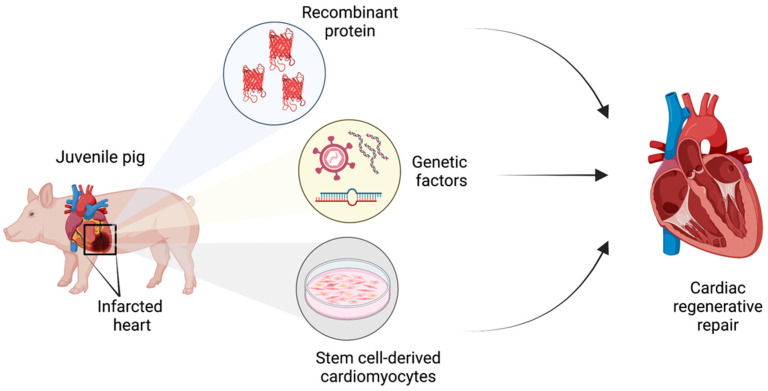
Schematic of therapeutic strategies utilized to induce cardiac regenerative repair in juvenile pig hearts following myocardial infarction.

Various therapeutic avenues have been explored utilizing swine as a large mammal model for human heart regenerative repair. In preclinical studies, porcine myocardial infarct models are utilized to cause rapid cardiomyocyte death in one region of the heart. The established methods for inducing myocardial infarction in pigs are: ischemia/reperfusion by temporary coronary occlusion [13,21], ischemia by permanent coronary occlusion [6,7], and coronary microembolization [22]. Following such injury, the extent of regenerative healing upon various experimental therapeutic strategies is then investigated. Broadly, the common strategies involve delivery of recombinant proteins to site of myocardial infarction to promote proliferative healing, induced expression or targeted depletion of genetic factors to promote cardiomyocyte proliferation, or the administration of cultured proliferative cardiomyocytes for engraftment to replace lost muscle (Figure 3), which are discussed in detail below.

### 3.1. Recombinant Protein Delivery

Factors involved in cardiomyocyte growth and development have been manipulated to assess preclinical therapeutic efficacy in the hearts of young swine after cardiac injury. The extracellular matrix protein agrin promotes cardiac regeneration in neonatal mice, and the potential benefit of local administration of agrin by retrograde delivery in coronary arteries after myocardial infarction (MI) was tested in three-month-old pigs [23,24]. A single dose of recombinant agrin administered during reperfusion after ischemia leads to increased cardiomyocyte survival, neovascularization, and improved cardiac function 28 days post-injury [24]. Increased proliferative activity of cardiomyocytes as detected by BrdU (Bromo-deoxyuridine) incorporation was also observed, along with decreased infarct size and increased vessel density. Since multiple beneficial effects were observed with agrin treatment, it is not clear that the improved cardiac function is the result of a proliferative response in cardiomyocytes. Similarly, the administration of the peptide growth factor Fstl1 via an epicardial patch showed improved cardiac function and reduced fibrotic remodeling after MI [25]. Cardiomyocyte cell cycling activity was monitored by EdU (5-ethynyl-2′-deoxyuridine) incorporation. Note that both BrdU and EdU are indicative of DNA synthesis, which could also represent endoreplication with increased number of nuclei per cardiomyocyte in swine. Thus, the delivery of these therapeutic agents is beneficial to the heart, but the evidence for cardiomyocyte proliferation or cardiac regeneration via the formation of new muscle in the context of adult cardiac injury is not clear in swine.

### 3.2. Gene Therapy

Multiple genetic mechanisms have been demonstrated to promote adult cardiomyocyte proliferation in mice. Recent efforts have been directed towards determining if these mechanisms are effective in promoting cardiomyocyte renewal in young adult pigs after injury. The development of adeno-associated virus (AAV) serotypes, such as AAV6 and AAV9, which infect porcine cardiomyocytes, has facilitated these gene therapy approaches that potentially could be translated to human patients.

MicroRNAs (miR) have been manipulated to induce cardiac regeneration in rodents [26], and miR-199a was identified as a therapeutic candidate for inducing cardiomyocyte proliferation and regeneration after MI. The ability to promote porcine cardiomyocyte proliferation after MI was tested by AAV6-mediated delivery of miR-199a in pigs [27]. The injection of AAV6-miR-199a into the left ventricles of 3–4-month-old male pigs after MI resulted in reduced scarring and improved cardiac function one month after injury. At 12 days after injury, multiple indicators of cardiomyocyte cell cycle activity were increased, including Ki67, BrdU incorporation, and pHH3+ nuclei. In addition, increased numbers of mononucleated cardiomyocytes were reported, with the caveat that nuclear number was assessed in intact tissue, in which it is difficult to accurately assess nucleation. Together, these data support the induction of cardiomyocyte proliferation by AAV6-mediated expression of miR-199a. Unfortunately, the long-term expression of miR-199a leads to cardiomegaly and sudden death in swine, underscoring the need to inactivate cardiac regenerative pathways long-term in a therapeutic setting.

Among the most effective ways to stimulate cardiomyocyte proliferation in neonatal and adult rodents is inactivation of the Hippo signaling pathway which controls organ size [28]. The potential therapeutic benefit of Hippo pathway inhibition in adult pigs after MI was tested with AAV9-mediated knockdown of the Hippo pathway gene Salvador via short hairpin (sh)RNA [29]. In this study, AAV9-*Sav*-shRNA was injected into the border zone of infarcted three-month-old pig hearts. At three months post-injection, AAV9-mediated Sav knockdown hearts exhibited improved cardiac function, less scarring, and improved vascularity compared to vector-injected controls. In pigs analyzed at 1-month post-treatment, mitotic activity assessed by EdU incorporation and pHH3 staining in cardiomyocytes, together with sarcomere breakdown and Aurora B kinase indicative of cytokinesis, were increased, providing evidence for cardiomyocyte proliferation and renewal after injury. Together, these studies support the possibility of an AAV9-mediated gene therapy approach to promote cardiomyocyte renewal in adult pig hearts through the targeted inhibition of the Hippo pathway.

### 3.3. Cell Therapy

Multiple stem/progenitor cell therapy approaches for cardiac regenerative repair have been tested in various mammalian model systems. The beneficial effects on cardiac recovery in most of these studies likely stem from paracrine or anti-inflammatory effects that promote cardiomyocyte survival and function after injury [30]. In one study utilizing Göttingen minipigs, upon administration of cortical bone stem cells (CBSC) post-MI, cardiac functional recovery and reduction in pathological ventricular remodeling was reported alongside significantly increased proliferative activity in cardiac non-myocytes [21]. However, in another study, partial remuscularization of the injured pig heart was reported, with newly differentiated graft myocardium observed after the implantation of large numbers (10^9^) of human embryonic stem cell (hESC)-derived cardiomyocytes post-MI [31]. Despite successful engraftment and remuscularization by hESC-derived cardiomyocytes, incorrect electrical coupling with pre-existing ventricular cardiomyocytes led to tachycardia in the weeks after implantation in swine. Similar severe arrhythmias were observed with hESC-derived cardiomyocyte engraftment studies in nonhuman primate hearts, which poses further challenges in translating this approach for human therapy [32]. Another proposed strategy for remuscularization of the diseased heart is the reprogramming of cardiac fibroblasts into cardiomyocyte-like cells [33]. Successful transdifferentiation of porcine cardiac fibroblasts into cardiomyocyte-like cells has been demonstrated in vitro utilizing lentiviral vectors expressing cardio-reprogramming factors (Gata4, Mef2c, Tbx5) alongside miR-590 [34]. However, whether this strategy will provide postinfarct recovery in vivo in swine has not been tested.

The regeneration of myocardium with induced pluripotent stem cell-derived cardiomyocytes (iPSC-CMs) has long been a goal in the field. However, the immature phenotype together with limited proliferative capacity of iPSC-CMs have so far impeded their use for in vivo cardiac therapeutics [35]. A strategy that has been adopted is combining genetic manipulations with iPSC-CMs to promote their proliferative potential for better healing in vivo. For example, human iPSC-CMs overexpressing the cell cycle gene Cyclin D2 (CCND2) were injected into injured myocardium in juvenile swine to test for the ability to engraft and repair [36]. Compared to the administration of wildtype iPSC-CMs, the CCND2-overexpressing iPSC-CMs lead to better functional recovery without apparent arrhythmias. However, while some increase in EdU incorporation was detected in both engrafted and resident cardiomyocytes, the main mechanism of action was determined to be paracrine effects promoting cardiomyocyte survival and angiogenesis, potentially through miR-mediated inhibition of the Hippo signaling pathway. These studies further support the existence of potentially beneficial paracrine factors with cardioprotective, not regenerative, activity in the setting of cardiac injury that warrants further investigation.

## 4. Advantages and Disadvantages of Porcine Models of Mammalian Heart Development and Regeneration

Definitive assessment of *de novo* generation of cardiomyocytes in pigs is limited by the lack of genetic reporters and lineage-tracing tools available in mice [37]. Thus, a thorough assessment of regenerative repair in pigs can be confounded by relative inability to distinguish between significant proliferative repair and regeneration versus functional recovery. In general, EdU or BrdU incorporation techniques or staining for Ki67 and Phosphohistone H3 mitotic markers have been utilized to detect cardiomyocyte cell cycling. A limitation of these assays is that they detect DNA synthesis and do not distinguish between the multinucleation, polyploidization of existing nuclei, or *de novo* generation of cardiomyocytes. Even when cytokinetic assays are performed, the identification of dividing cardiomyocytes with characteristic post-abscission midbodies indicative of successful cytokinesis can be challenging, especially in histological sections. An additional caveat of cardiac regenerative studies in pigs and rodents is that the robust induction of cardiomyocyte cell cycle activity can lead to cardiac dysfunction in the long term [27,38]. Thus, it is necessary to be able to control both induction and inactivation of cardiomyocyte proliferative responses in cardiac regenerative/reparative therapies.

The multinucleation and growth characteristics of cardiomyocytes are very different among mammals, including mice, pigs, and humans [15,18]. Typically, due to the high cost of pig husbandry and convenience, pigs at 2–4 months of postnatal age are utilized for preclinical cardiac studies [2]. However, at 2–4 postnatal months, swine are still at the early juvenile stages of development. At this stage, cardiomyocyte cell cycling resulting in increased multinucleation as well as the transition to hypertrophic mode of growth are still ongoing [3]. An additional consideration is that traditionally used white Yorkshire farm pigs continue to grow for several years after sexual maturity, reaching up to 750 pounds for males. Thus, Göttingen minipigs have been developed as a research strain to facilitate physiological and interventional analyses of heart regeneration and repair [39]. While careful analysis of cardiomyocyte growth characteristics has not been performed in minipigs, multinucleation of individual cardiomyocytes has been observed [21]. Thus, the impact of multinucleation and longitudinal growth dynamics of cardiomyocytes on the outcome of various regenerative strategies being tested in juvenile pigs needs to be considered.

Despite such challenges, several of the regenerative therapeutics that have been preclinically tested in swine do provide evidence of beneficial effects on cardiac performance and myocardial integrity after cardiac injury (Table 1) [21,24,29,36]. Treatment of infarcted porcine hearts with various cardioprotective factors or cell- and gene-based therapies remains an attractive preclinical model for the development of cardiac therapies for human cardiovascular disease. While pleiotropic and paracrine effects appear to be guiding such improvements in cardiac function for the most part, investigating the mechanisms that underlie such benefits in swine can facilitate clinical translation to human hearts.

At the organ level, human and pig cardiac physiology is sufficiently similar for the preclinical testing of mechanical or surgical innovations for treating human heart disease. Due to the great similarities in the anatomy and function of juvenile swine and human hearts, such as similar heart size, body weight, and cardiac load, swine are often utilized for preclinical tests of biomechanical devices, with great success [40]. Further, swine may prove an excellent resource for developing xenotransplantation tools for curing heart disease. A report in 2018 showed promising results for cardiac xenotransplantation from pigs to nonhuman primates [41], with the successful maintenance of a pig donor heart in a baboon host for over six months. To successfully evade xenograft rejection and donor organ overgrowth in baboon host, genetic knockout strategies to suppress organ rejection in tandem with temsirolimus treatment to control cardiac growth were utilized. Pigs with genetic modifications to knockout the growth hormone receptor as a way to control donor organ overgrowth are also being generated and tested [42]. Most recently, in early 2022, the heart from a genetically modified pig with combined immunosuppression and growth hormone pathway ablation was used as a source for cardiac xenotransplantation in a human clinical study [43]. Despite promising early outcomes, however, the human recipient died two months after surgery, and the underlying mechanisms are currently under study [44]. It thus remains to be seen whether such surgical interventions utilizing genetically modified juvenile pig hearts can become a key therapeutic strategy for the failing human heart in the next decade.

## 5. Conclusions and Future Perspectives

Pigs have been popular as preclinical models for investigation of new therapies for translation to human disease. However, despite an increasing number of reports of successful preclinical testing of drugs, tissue-engineered patches, AAV-mediated gene therapies, and cell-based therapies designed to promote cardiac repair after injury, none of these approaches have been safely and efficaciously translated into the clinic for healing the human heart. In this review, we discuss the proliferative and regenerative potential of cardiomyocytes in neonatal pigs alongside preclinical therapeutic strategies for inducing cardiac regenerative repair in juvenile pig injury models. While pigs and rodents exhibit a similar early neonatal period of transient regenerative capacity, an early neonatal window of cardiac regenerative capacity in human infants has not been definitively demonstrated. However, there is potential for the discovery of novel factors for inducing cardiac regeneration in humans using the porcine model, especially when taken together with studies in rodents.

A limitation of the pig model is that cardiomyocyte maturational dynamics and growth mechanisms are distinct in swine compared to other mammals studied so far. Unlike humans or rodents, mature cardiomyocytes in juvenile and adult swine are highly multinucleated, which could limit the translation of results when older swine are utilized as preclinical models for regenerative therapeutic strategies. Thus, increased rigor in identifying *de novo* cardiomyocyte generation, as opposed to pleiotropic beneficial effects that result in cardiac functional recovery post-injury, is necessary to accurately interpret cardiac regenerative strategies in swine for clinical translation to human disease. Still, pigs remain an attractive model for preclinical surgical and interventional approaches due to their similarities with human hearts in terms of anatomy and physiology. With the advent of gene targeting and recent advances in xenotransplantation, the use of the pig system for studies of cardiac regeneration and repair is likely to accelerate in the years to come.

## Figures and Tables

**Table 1:** Summary of porcine developmental and therapeutic heart regeneration studies (References in text).

Regenerative Strategy	Therapeutic Candidate	Porcine Model	Postnatal Age	Cardiomyocyte Proliferation	Functional Recovery
Neonatal Injury (Innate Regenerative Capacity)	Unknown	Yorkshire	0 to 2 days	Yes; Self-limiting	Yes
	Unknown	Yorkshire	30 days	None	No
Recombinant Protein Delivery	Agrin	German	60 days	Unclear	Yes
	Fstl1	Yorkshire	45 days	Unclear	Yes
AAV-mediated Gene Therapy	miR-199a	Yorkshire	3 to 4 months	Yes; Uncontrolled	Yes; Temporary
	shSalvador	Yorkshire	3 months	Yes	Yes
Cell Therapy	Cortical Bone SCs	Göttingen mini	9 to 12 months	Unclear	Yes
	hESC-derived CMs	Yorkshire	Adult	Unclear	Unclear
	Gene-edited human iPSC-derived CMs	Yorkshire	2 months	Unclear	Yes

## References

[1] Crespo-Leiro M.G., Metra M., Lund L.H., Milicic D., Costanzo M.R., Filippatos G.F., Gustafsson S., Tsui E., Barge-Caballero N., De Jonge M. (2018). Advanced heart failure: A position statement of the Heart Failure Association of the European Society of Cardiology. Eur. J. Heart Fail..

[2] Camacho P., Fan H., Liu Z., He J.Q. (2016). Large Mammalian Animal Models of Heart Disease. J. Cardiovasc. Dev. Dis..

[3] Velayutham N., Alfieri C.M., Agnew E.J., Riggs K.W., Baker R.S., Ponny S.R., Zafar F., Yutzey K.E. (2020). Cardiomyocyte cell cycling, maturation, and growth by multinucleation in postnatal swine. J. Mol. Cell Cardiol..

[4] Vivien C.J., Hudson J.E., Porrello E.R. (2016). Evolution, comparative biology and ontogeny of vertebrate heart regeneration. NPJ Regen Med..

[5] Porrello E.R., Mahmoud A.I., Simpson E., Hill J.A., Richardson J.A., Olson E.N., Sadek H.A. (2011). Transient regenerative potential of the neonatal mouse heart. Science.

[6] Ye L., D’Agostino G., Loo S.J., Wang C.X., Su L.P., Tan S.H., Tee G.Z., Pua C.J., Pena E.M., Cheng R.B. (2018). Early Regenerative Capacity in the Porcine Heart. Circulation.

[7] Zhu W., Zhang E., Zhao M., Chong Z., Fan C., Tang Y., Hunter J.D., Borovjagin A.V., Walcott G.P., Chen J.Y. (2018). Regenerative Potential of Neonatal Porcine Hearts. Circulation.

[8] Saker D.M., Walsh-Sukys M., Spector M., Zahka K.G. (1997). Cardiac recovery and survival after neonatal myocardial infarction. Pediatr. Cardiol..

[9] Haubner B.J., Schneider J., Schweigmann U., Schuetz T., Dichtl W., Velik-Salchner C., Stein J.I., Penninger J.M. (2016). Functional Recovery of a Human Neonatal Heart After Severe Myocardial Infarction. Circ. Res..

[10] Mollova M., Bersell K., Walsh S., Savla J., Das L.T., Park S.Y., Silberstein L.E., Dos Remedios C.G., Graham D., Colan S. (2013). Cardiomyocyte proliferation contributes to heart growth in young humans. Proc. Natl. Acad. Sci. USA.

[11] Bergmann O., Zdunek S., Felker A., Salehpour M., Alkass K., Bernard S., Sjostrom S.L., Szewczykowska M., Jackowska T., Dos Remedios C. (2015). Dynamics of Cell Generation and Turnover in the Human Heart. Cell.

[12] Bergmann O., Bhardwaj R.D., Bernard S., Zdunek S., Barnabe-Heider F., Walsh S., Zupicich J., Alkass K., Buchholz B.A., Druid H. (2009). Evidence for cardiomyocyte renewal in humans. Science.

[13] Agnew E.J., Velayutham N., Matos Ortiz G., Alfieri C.M., Hortells L., Moore V., Riggs K.W., Baker R.S., Gibson A.M., Ponny S.R. (2019). Scar Formation with Decreased Cardiac Function Following Ischemia/Reperfusion Injury in 1 Month Old Swine. J. Cardiovasc. Dev. Dis..

[14] Zhao M., Zhang E., Wei Y., Zhou Y., Walcott G.P., Zhang J. (2020). Apical Resection Prolongs the Cell Cycle Activity and Promotes Myocardial Regeneration After Left Ventricular Injury in Neonatal Pig. Circulation.

[15] Velayutham N., Agnew E.J., Yutzey K.E. (2019). Postnatal Cardiac Development and Regenerative Potential in Large Mammals. Pediatr. Cardiol..

[16] Guo Y., Pu W.T. (2020). Cardiomyocyte Maturation: New Phase in Development. Circ. Res..

[17] Patterson M., Barske L., Van Handel B., Rau C.D., Gan P., Sharma A., Parikh S., Denholtz M., Huang Y., Yamaguchi Y. (2017). Frequency of mononuclear diploid cardiomyocytes underlies natural variation in heart regeneration. Nat. Genet..

[18] Hirose K., Payumo A.Y., Cutie S., Hoang A., Zhang H., Guyot R., Lunn D., Bigley R.B., Yu H., Wang J. (2019). Evidence for hormonal control of heart regenerative capacity during endothermy acquisition. Science.

[19] Liu H., Zhang C.H., Ammanamanchi N., Suresh S., Lewarchik C., Rao K., Uys G.M., Han L., Abrial M., Yimlamai D. (2019). Control of cytokinesis by beta-adrenergic receptors indicates an approach for regulating cardiomyocyte endowment. Sci. Transl. Med..

[20] Han L., Choudhury S., Mich-Basso J.D., Ammanamanchi N., Ganapathy B., Suresh S., Khaladkar M., Singh J., Maehr R., Zuppo D.A. (2020). Lamin B2 Levels Regulate Polyploidization of Cardiomyocyte Nuclei and Myocardial Regeneration. Dev. Cell.

[21] Sharp T.E., Schena G.J., Hobby A.R., Starosta T., Berretta R.M., Wallner M., Borghetti G., Gross P., Yu D., Johnson J. (2017). Cortical Bone Stem Cell Therapy Preserves Cardiac Structure and Function After Myocardial Infarction. Circ. Res..

[22] Moller-Helgestad O.K., Ravn H.B., Moller J.E. (2018). Large Porcine Model of Profound Acute Ischemic Cardiogenic Shock. Methods Mol. Biol..

[23] Bassat E., Mutlak Y.E., Genzelinakh A., Shadrin I.Y., Baruch Umansky K., Yifa O., Kain D., Rajchman D., Leach J., Riabov Bassat D. (2017). The extracellular matrix protein agrin promotes heart regeneration in mice. Nature.

[24] Baehr A., Umansky K.B., Bassat E., Jurisch V., Klett K., Bozoglu T., Hornaschewitz N., Solyanik O., Kain D., Ferraro B. (2020). Agrin Promotes Coordinated Therapeutic Processes Leading to Improved Cardiac Repair in Pigs. Circulation.

[25] Wei K., Serpooshan V., Hurtado C., Diez-Cunado M., Zhao M., Maruyama S., Zhu W., Fajardo G., Noseda M., Nakamura K. (2015). Epicardial FSTL1 reconstitution regenerates the adult mammalian heart. Nature.

[26] Eulalio A., Mano M., Dal Ferro M., Zentilin L., Sinagra G., Zacchigna S., Giacca M. (2012). Functional screening identifies miRNAs inducing cardiac regeneration. Nature.

[27] Gabisonia K., Prosdocimo G., Aquaro G.D., Carlucci L., Zentilin L., Secco I., Ali H., Braga L., Gorgodze N., Bernini F. (2019). MicroRNA therapy stimulates uncontrolled cardiac repair after myocardial infarction in pigs. Nature.

[28] Liu S., Martin J.F. (2019). The regulation and function of the Hippo pathway in heart regeneration. Wiley Interdiscip. Rev. Dev. Biol..

[29] Liu S., Li K., Wagner Florencio L., Tang L., Heallen T.R., Leach J.P., Wang Y., Grisanti F., Willerson J.T., Perin E.C. (2021). Gene therapy knockdown of Hippo signaling induces cardiomyocyte renewal in pigs after myocardial infarction. Sci. Transl. Med..

[30] Vagnozzi R.J., Maillet M., Sargent M.A., Khalil H., Johansen A.K.Z., Schwanekamp J.A., York A.J., Huang V., Nahrendorf M., Sadayappan S. (2020). An acute immune response underlies the benefit of cardiac stem cell therapy. Nature.

[31] Romagnuolo R., Masoudpour H., Porta-Sanchez A., Qiang B., Barry J., Laskary A., Qi X., Masse S., Magtibay K., Kawajiri H. (2019). Human Embryonic Stem Cell-Derived Cardiomyocytes Regenerate the Infarcted Pig Heart but Induce Ventricular Tachyarrhythmias. Stem. Cell Rep..

[32] Chong J.J., Yang X., Don C.W., Minami E., Liu Y.W., Weyers J.J., Mahoney W.M., Van Biber B., Cook S.M., Palpant N.J. (2014). Human embryonic-stem-cell-derived cardiomyocytes regenerate non-human primate hearts. Nature.

[33] Addis R.C., Ifkovits J.L., Pinto F., Kellam L.D., Esteso P., Rentschler S., Christoforou N., Epstein J.A., Gearhart J.D. (2013). Optimization of direct fibroblast reprogramming to cardiomyocytes using calcium activity as a functional measure of success. J. Mol. Cell Cardiol..

[34] Singh V.P., Mathison M., Patel V., Sanagasetti D., Gibson B.W., Yang J., Rosengart T.K. (2016). MiR-590 Promotes Transdifferentiation of Porcine and Human Fibroblasts Toward a Cardiomyocyte-Like Fate by Directly Repressing Specificity Protein 1. J. Am. Heart Assoc..

[35] Mummery C.L. (2018). Perspectives on the Use of Human Induced Pluripotent Stem Cell-Derived Cardiomyocytes in Biomedical Research. Stem. Cell Rep..

[36] Zhao M., Nakada Y., Wei Y., Bian W., Chu Y., Borovjagin A.V., Xie M., Zhu W., Nguyen T., Zhou Y. (2021). Cyclin D2 Overexpression Enhances the Efficacy of Human Induced Pluripotent Stem Cell-Derived Cardiomyocytes for Myocardial Repair in a Swine Model of Myocardial Infarction. Circulation.

[37] Auchampach J., Han L., Huang G.N., Kuhn B., Lough J.W., O’Meara C.C., Payumo A.Y., Rosenthal N.A., Sucov H.M., Yutzey K.E. (2022). Getting it right: Measuring cardiomyocyte cell cycle activity and proliferation in the age of heart regeneration. Am. J. Physiol. Heart Circ. Physiol..

[38] D’Uva G., Aharonov A., Lauriola M., Kain D., Yahalom-Ronen Y., Carvalho S., Weisinger K., Bassat E., Rajchman D., Yifa O. (2015). ERBB2 triggers mammalian heart regeneration by promoting cardiomyocyte dedifferentiation and proliferation. Nat. Cell Biol..

[39] Schuleri K.H., Boyle A.J., Centola M., Amado L.C., Evers R., Zimmet J.M., Evers K.S., Ostbye K.M., Scorpio D.G., Hare J.M. (2008). The adult Gottingen minipig as a model for chronic heart failure after myocardial infarction: Focus on cardiovascular imaging and regenerative therapies. Comp. Med..

[40] Ouyang H., Liu Z., Li N., Shi B., Zou Y., Xie F., Ma Y., Li Z., Li H., Zheng Q. (2019). Symbiotic cardiac pacemaker. Nat. Commun..

[41] Langin M., Mayr T., Reichart B., Michel S., Buchholz S., Guethoff S., Dashkevich A., Baehr A., Egerer S., Bauer A. (2018). Consistent success in life-supporting porcine cardiac xenotransplantation. Nature.

[42] Goerlich C.E., Griffith B., Hanna P., Hong S.N., Ayares D., Singh A.K., Mohiuddin M.M. (2021). The growth of xenotransplanted hearts can be reduced with growth hormone receptor knockout pig donors. J. Thorac. Cardiovasc. Surg..

[43] Reardon S. (2022). First pig-to-human heart transplant: What can scientists learn?. Nature.

[44] Rabin R.C. (2022). Patient in Groundbreaking Heart Transplant Dies. The New York Times.

